# Factors associated with the implementation of the 5As model of smoking cessation support during pregnancy: A scoping review

**DOI:** 10.18332/tid/169623

**Published:** 2023-08-30

**Authors:** Adrianna Burtin, Estelle Clet, Nolwenn Stevens, Charlotte Kervran, Manon Frevol, Rébecca Ratel, Perrine Moysan, François Alla, 5A-QUIT-N group

**Affiliations:** 1 Department of Methodology and Innovation in Prevention, Bordeaux University Hospital, Bordeaux, France; 2 Inserm UMR 1219-Bordeaux Population Health, University of Bordeaux, Bordeaux, France

**Keywords:** smoking cessation, 5As method, health promotion, prenatal care, pregnancy

## Abstract

**INTRODUCTION:**

The prevalence of smoking among pregnant women is the highest in the European region, making smoking cessation a public health priority. In order to address this, pregnant smokers need to be better supported by their healthcare professionals in their attempts to quit smoking. The 5As model, which is a psychosocial intervention, seems to be effective in this specific population. The objective of this review is to identify the factors that act as barriers or facilitators to the implementation of the 5As model within prenatal practices.

**METHODS:**

We conducted a scoping review of the literature on PubMed and Scopus databases, using the terms: ‘smoking cessation’, ‘pregnan*’, and (‘5A’ or ‘5As’). The identified factors were categorized using a theoretical framework of The European Observatory on Health Systems and Policies.

**RESULTS:**

Among the 43 articles identified in the databases, 13 articles were included in this review. In total, we identified 48 factors. When necessary, we grouped them together, resulting in 12 sub-categories, which in turn were grouped into 9 categories. Those 9 categories were then classified into the 3 levels of the theoretical framework: the clinical level (motivation), the organizational level (healthcare pathway), and the health system level (political environment).

**CONCLUSIONS:**

The factors identified are varied and numerous and are involved in each level of the theoretical framework.

## INTRODUCTION

Smoking is the second largest risk factor for early death and associated morbidity^1,2^. Some populations are more sensitive to its harmful effects. This is notably the case for pregnant smokers, in whom smoking is one of the main avoidable causes of pregnancy complications, particularly for the newborn child^3^. In fact, smoking during pregnancy is associated with an excess risk of gestational diabetes, lung infection and extra-uterine pregnancies in women^3-5^, and with delayed intrauterine growth, central nervous system toxicity, sudden infant death, respiratory problems, and congenital defects in children. Children exposed to smoking are also at increased risk of becoming smokers during their lifetime^6-8^.

The prevalence of smoking among pregnant women is the highest in the European region^9^, with about 12.5% in Austria, 16.3% in France and 12–17% in the UK^10^. Pregnancy, a key event in a woman’s life^11^, can be considered to be a ‘teachable moment’, i.e. a moment when women are more likely to change their health behaviors^12^. This period is, therefore, particularly effective for accompanying women in the reduction of tobacco consumption. However, effective smoking cessation strategies applied to the general population appear to have little effect on pregnant women^13-15^. Therefore, the factors associated with the implementation of strategies remain to be clarified, particularly in this population.

The highly addictive nature of nicotine in tobacco products makes quitting particularly difficult and often requires additional support from a health professional (HP)^16^. Psychosocial interventions put in place by these professionals appear to be the most recommended for pregnant women, including the 5As model^13,17^. In pregnant women, the 5As model seems to be effective if the intervention is context sensitive^18-22^. This model is supported by a high level of evidence^23^ and aims to identify the smoking status and encourage cessation by proposing strategies adapted to the individual’s degree of motivation^24^. The 5As method includes five steps: Ask if the woman smokes; Advise her to stop smoking; Assess her motivation, the severity of her addiction and her social environment; Assist to put in place strategies to help her quit smoking; and Arrange follow-up visits throughout the intervention^23,24^. The 5As model can therefore help coordinate different effective smoking cessation strategies for pregnant women. For example, the ‘Ask’ step can be done using a self-administered questionnaire, the ‘Advise’ step can be done by giving a pamphlet about the benefits of smoking cessation, the ‘Assess’ step can be done using some tools to assess the predictors of smoking cessation, such as the Fagerström test for nicotine dependence^25^, the ‘Assist’ step can be done prescribing nicotine replacement therapy, and the ‘Arrange’ step can be done using the electronic medical record which can be set to remind the health professional to ask the pregnant women about tobacco.

This type of intervention is complex due to the large number of components involved and the different levels of involvement (e.g. pregnant women, health professionals, local healthcare organizations, and political and legal context)^26^. The effectiveness of the 5As model in terms of public health depends not only on the five key steps of the intervention but also on the contextual factors in which it is implemented^27,28^. This article hopes to assist in operationalizing the 5As model within prenatal practice by reviewing studies exploring experiences with pregnant smokers. The objective of this review is to identify the factors that act as barriers or facilitators to the implementation of the 5As model in the health system.

## METHODS

A scoping review of the literature following the Arksey and O’Malley methodological framework^29^ and the PRISMA 2020 statement, which proposes a structured approach with a four-phase flow diagram, and a 27-item checklist^30^ was conducted (Supplementary file).

### Eligibility criteria

Articles were retained if they were in English or French, published between January 2010 and 2021, and concerned pregnant women and the 5As model. We have included all types of settings, quantitative and qualitative research studies, and research designs. Articles detailing the 5As model without mentioning its implementation were excluded. Thus, protocols, guidelines and recommendations were not included.

### Information sources and search strategy

In February 2023, we searched PubMed, Scopus and Web of Science, using the terms: ‘smoking cessation’, ‘pregnan*’ and (‘5A’ or ‘5As’) in titles and abstracts.

### Selection process and data collection process

The lists of articles found in both databases were manually compared to remove any duplicates. The two reviewers (EC and AB) independently read all the abstracts of the articles found. Articles were excluded if they did not meet the inclusion criteria. Thus, eligible articles were selected on the basis of their abstract by two reviewers (EC and AB) and then were documented in a Microsoft Excel spreadsheet. This document was uploaded on the Bordeaux university hospital’s secure platform, Nextcloud. A kappa statistic was used to measure the correlation between the two reviewers for the abstract screening. Any discrepancies were discussed between the reviewers until a consensus was reached.

The two reviewers then performed a full-text assessment to identify the relevant articles to include in the review. The full articles were analyzed using a grid specifically developed for the study, identifying several elements: authors, title, date, where the study was conducted, and the method used. Three articles were assessed by the two reviewers independently to ensure that they were analyzing the articles in a similar way. During the full-text assessment step, if a study did not meet all of the inclusion criteria, it was excluded. The reviewers then independently identified the factors associated with the implementation of the 5As model present in each study.

### Data analysis

The factors identified in the reviews during data extraction were classified by the reviewers into thematic sub-categories and then into thematic categories. These categories and sub-categories were developed inductively, based on the data and specifically for this study. To go further in the classification, we used a theoretical framework from the European Observatory on Health Systems and Policies to organize each of the factors into three levels: a clinical level, an organizational level, and a health systems level^31^. Thus, the thematic categories of factors have also been classified into the three categories of the theoretical model above.

## RESULTS

**Figure 1:**
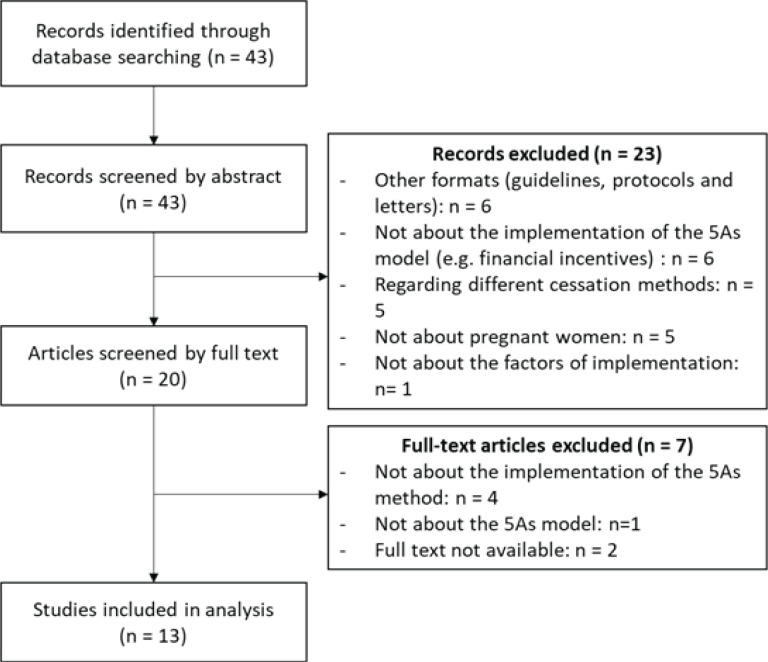
PRISMA flow chart

A total of 43 articles were identified. Among them, 13 met the inclusion criteria^32-44^ (Figure 1). The majority of the articles came from English-speaking countries (Supplementary file Material 1), mainly the USA, Australia and South Africa (n=10; 76.9%). The 5As method is used in many contexts, such as maternal obstetric units, prenatal care clinics, and in the public health system in general.

A thematic analysis of the 13 articles allowed us to identify the factors associated with the implementation of the 5As model. These factors are detailed in the factors associated with the implementation of the 5As model section (Supplementary file Material 2). In total, we identified 48 factors. When necessary, we grouped them together, resulting in 12 sub-categories, which in turn were grouped into 9 categories. The 9 categories are: clinical practices, organization of practices, resources, support for professionals, perceptions of the 5As model, professional role and identity, health professionals’ perceptions of pregnant women, influence of beneficiaries, and the political environment. These 9 categories were then classified into the 3 levels of the theoretical framework described above.

### Clinical level

*Clinical practices*

‘Clinical practices’ refers to the target practices for professionals implementing the 5As model. The category of clinical practices has three sub-categories. Firstly, six studies (46.2%) emphasized the importance of the professional’s approach, attitudes, posture and professional relationship with women in their care^34-37,43,44^. Similarly, four studies (30.8%) highlighted that interprofessional cooperation as an important element in the successful implementation of the 5As model^35-37,41^. The last sub-category that was identified by seven studies (53.9%) was the use of practical aids such as motivational interviewing or smoking cessation information materials^33-36,38,41,43^.

*Perceptions of the 5As model*

This category takes into account the perceptions of perinatal professionals on the practice of the 5As model. It was not necessary to create subcategories here. Four of the thirteen studies included in this scoping review discussed this theme (30.8%)^34,38,40,41^.

*Professional role and identity*

The next category explores the role and identity of perinatal professionals in supporting pregnant women to stop smoking. Three sub-categories were identified. The first is the role perceived by the professionals themselves, which was identified in five studies (38.5%)^35,36,40,43,44^. The second sub-category is the motivation of these professionals, which also appeared in five studies (38.5%)^35,38,39,42,44^. Finally, the last sub-category to be considered for the implementation of the 5As model is the self-efficacy of professionals. This was cited by seven studies (53.9%)^32,34,35,40,41,43,44^.

*Health professionals’ perceptions of pregnant women*

Another theme that was identified in our thematic analysis was the perceptions that perinatal professionals have of the pregnant smokers for whom they provide care. It may be the professionals’ preconceived notions of pregnant women. This is an element that was mentioned in seven of the thirteen studies in this review (53.9%)^34,35,38,39,41,43,44^. This category did not require sub-categorization.

*Influence of beneficiaries*

This theme deals with the influence of the pregnant woman (the beneficiary) on the implementation of the 5As model by perinatal professionals. It was found in three studies (23.1%)^35,41,43^. There is no subcategory.

### Organization level

*Organization of practices*

These are the organizational factors required for a successful implementation of the 5As model. This second category has two sub-categories which are the organization of the perinatal care pathway, identified in five studies (38.5%)^34,35,39,41,44^, and the organization of the deployment of the 5As model, also cited by four studies (30.8%)^35,36,39,41^.

*Resources*

These are the resources needed to support the implementation of the 5As model. Financial resources and time are the two types of resources mentioned in the literature and therefore constitute the two sub-categories. The issue of financial resources was raised by five studies (38.5%)^34,35,37,40,42^ and time by eleven studies (84.6%)^34-44^. Indeed, the majority of the included studies highlighted the lack of time available for professionals in general and during consultations as a barrier to the implementation of the 5As model.

*Support for professionals*

The next category concerns support for professionals who wish to implement the 5As model with their patients. Here we see that the training of professionals is an important element. Indeed, all the included studies (100%) stressed the importance of training and the acquisition of appropriate skills and knowledge^32-44^. The second sub-category concerns the resources available to support professionals in the practice of the 5As model. Of the thirteen included studies, six (46.2%) raised the importance of these resources^33,35-37,39,41^.

### Health system level

*Political environment*

The last category concerns the political context in which the 5As model is deployed. It does not have a subcategory. This political context is an element that was found in three articles (23.1%)^34-36^.

## DISCUSSION

To the best of our knowledge, this is the first scoping review that focuses on the factors associated with the implementation of the 5As model for pregnant women. Our review identified 49 factors, classified into three different levels: a clinical level, an organizational level, and a health system level. The 5As model is a complex intervention, because ‘of properties of the intervention itself, such as the number of components involved; the range of behaviors targeted; expertise and skills required by those delivering and receiving the intervention; the number of groups, settings, or levels targeted; or the permitted level of flexibility of the intervention or its components’^45^.

Consequently, several elements must be studied prior to implementation, particularly the contextual elements, since these are in constant interaction with the intervention and are highly influential in the way the intervention is implemented and received. This creates what is known as the ‘interventional system’^27^ because it takes into account the influence that the environment has on the intervention at the local level^28^. In the context of the implementation of the 5As model, the environment to be considerate of is the health system, whose functioning changes depending on the region or territory (medical density, territorial organization, etc.). The results of this review highlight the need to take into account the three levels of the interventional system, as they are each influential in the implementation of a 5As intervention^31^. For example, although the training of professionals is frequently emphasized, it is not the only factor that can be mobilized; the majority of the factors identified in this review are organizational. Therefore, mobilizing several factors at all levels of the interventional system would lead to a more robust implementation of the 5As model^46,47^.

The complexity of the 5As model does not lie exclusively in the model itself. Indeed, much of this complexity is due to the context in which this model is implemented^28^. An intervention such as the implementation of the 5As model may be effective in one context but not in another. Adaptation to the context is thus paramount for the intervention to be effective^28,47^. The factors identified in this review are not context-specific, allowing those interested in implementing the 5As method to identify themselves the factors needed for success in their own environment. This enables the intervention to be flexible and adaptable to different environments and local health system organizations^47^.

In addition, the nature of the factors identified by this literature review is consistent with pre-existing theoretical frameworks. For example, the theoretical framework proposed by Senn et al.^48^ also highlights the diversity of domains influencing practice in primary care (patient and population needs, organization and structure of primary care practices, patient and population health outcomes, and delivery of primary care services). The results of this review are consistent with previous studies observing smoking cessation interventions. Indeed, several studies^49-51^ have demonstrated the importance of individual factors (training, personal experiences, self-efficacy, smoking status, etc.) as well as organizational factors (knowledge/existence of guidelines/resources, organizational support, leadership, etc.) in the implementation of smoking cessation advice by health professionals. Finally, the factors for such a cessation method are numerous and varied. To implement a 5As model intervention, it will be necessary to take into account the many different elements that can influence the intervention.

### Strengths and limitations

This study has several strengths and limitations. Some factors may be missing from this report as they have not yet been published for one of several reasons: some experiments of the 5As model may not be documented in written reports, some may be published in the grey literature, and others may be published as indexed articles in non-healthcare databases (e.g. implementation sciences, management sciences). The risk of missing factors is estimated to be low, given the diversity of the selected studies. This diversity ensures that many aspects of the implementation of the 5As model have been explored. Moreover, because of the rigorous method used for this scoping review, we believe we have identified a complete list of the factors that appear in published articles. Furthermore, the databases PubMed, Scopus, and Web of Science were used for this review, which are some of the main reference databases in the health field.

However, it remains impossible to know whether certain factors must be present for the implementation to be effective. While some factors are found every time in the articles and others are found only once, this does not necessarily mean that those that are always present are indispensable and those that are less present are optional. Several conclusions can be drawn from this observation. Further studies are needed in order to determine which factors are truly essential to the implementation of the 5As model. This limitation of our study demonstrates the importance of adapting the intervention to the context by studying what are the specific weaknesses of the environment and how to respond to them.

Finally, because the 5As model is a complex intervention, all the factors identified for a successful 5As intervention are therefore interdependent. This study did not allow us to determine what levels of influence exist between them. For example, we know that the *influence of beneficiaries* and the *support for professionals* will have an influence on the *perceptions of pregnant women*, but we do not know if one has a stronger influence than the other. Further studies exploring these different links are needed to understand the influence pathways and to ensure that all the elements necessary for effective implementation are gathered and prioritized.

### Implications

Prior to the implementation, it is necessary to study the factors associated with the implementation of the 5As model in order to adapt to the singularities of each context. Further studies are needed to investigate whether certain factors must be present to guarantee the effectiveness of the implementation or simply have a facilitating role, as well as to identify the chains of influence between the factors.

## CONCLUSIONS

There are many factors associated with the implementation of the 5As model, and a selection must be made to ensure effective implementation. Several factors must be taken into account when selecting which to prioritize: the context in which the implementation takes place, the different levels of organization of the health system, and the existing resources. However, further studies are needed to investigate whether certain factors must be present to guarantee the effectiveness of the implementation or simply have a facilitating role, as well as to identify the pathways of influence between the factors.

## Data Availability

Data sharing is not applicable to this article as no new data was created.

## References

[1] GBD 2015 Risk Factors Collaborators (2016). Global, regional, and national comparative risk assessment of 79 behavioural, environmental and occupational, and metabolic risks or clusters of risks, 1990-2015: a systematic analysis for the Global Burden of Disease Study 2015. Lancet.

[2] GBD (2017). Tobacco Collaborators. Lancet.

[3] Mund M, Louwen F, Klingelhoefer D, Gerber A (2013). Smoking and pregnancy--a review on the first major environmental risk factor of the unborn. Int J Environ Res Public Health.

[4] Pineles BL, Park E, Samet JM (2014). Systematic review and meta-analysis of miscarriage and maternal exposure to tobacco smoke during pregnancy. Am J Epidemiol.

[5] Centers for Disease Control and Prevention (US), National Center for Chronic Disease Prevention and Health Promotion (US), Office on Smoking and Health (US) (2010). How Tobacco Smoke Causes Disease: The Biology and Behavioral Basis for Smoking-Attributable Disease: A Report of the Surgeon General.

[6] Taylor JA, Sanderson M (1995). A reexamination of the risk factors for the sudden infant death syndrome. J Pediatr.

[7] Ko TJ, Tsai LY, Chu LC (2014). Parental smoking during pregnancy and its association with low birth weight, small for gestational age, and preterm birth offspring: a birth cohort study. Pediatr Neonatol.

[8] Hackshaw A, Rodeck C, Boniface S (2011). Maternal smoking in pregnancy and birth defects: a systematic review based on 173 687 malformed cases and 11.7 million controls. Hum Reprod Update.

[9] Lange S, Probst C, Rehm J, Popova S (2018). National, regional, and global prevalence of smoking during pregnancy in the general population: a systematic review and meta-analysis. Lancet Glob Health.

[10] Euro-Peristat Project (2018). European Perinatal Health Report: core indicators of the health and care of pregnant women and babies in Europe in 2015.

[11] Cooper S, Orton S, Leonardi-Bee J (2017). Smoking and quit attempts during pregnancy and postpartum: a longitudinal UK cohort. BMJ Open.

[12] Lawson PJ, Flocke SA (2009). Teachable moments for health behavior change: a concept analysis. Patient Educ Couns.

[13] Chamberlain C, O’Mara-Eves A, Oliver S (2013). Psychosocial interventions for supporting women to stop smoking in pregnancy. Cochrane Database Syst Rev.

[14] Lumley J, Chamberlain C, Dowswell T, Oliver S, Oakley L, Watson L (2009). Interventions for promoting smoking cessation during pregnancy. Cochrane Database Syst Rev.

[15] Stead LF, Lancaster T (2012). Combined pharmacotherapy and behavioural interventions for smoking cessation. Cochrane Database Syst Rev.

[16] World Health Organization Framework Convention on Tobacco Control (2003). Convention–Cadre De L’oms Pour La Lutte Antitabac.

[17] Patnode CD, Henderson JT, Thompson JH, Senger CA, Fortmann SP, Whitlock EP (2015). Behavioral Counseling and Pharmacotherapy Interventions for Tobacco Cessation in Adults, Including Pregnant Women: A Review of Reviews for the U.S. Preventive Services Task Force.

[18] Fiore MC, Jaén CR, Baker TB (2008). Treating Tobacco Use and Dependence: 2008 Update.

[19] Zwar N, Richmond R, Borland R (2011). Supporting smoking cessation: a guide for health professionals.

[20] Melvin CL, Dolan-Mullen P, Windsor RA, Whiteside HP, Goldenberg RL (2000). Recommended cessation counselling for pregnant women who smoke: a review of the evidence. Tob Control.

[21] Olaiya O, Sharma AJ, Tong VT (2015). Impact of the 5As brief counseling on smoking cessation among pregnant clients of Special Supplemental Nutrition Program for Women, Infants, and Children (WIC) clinics in Ohio. Prev Med.

[22] Bar-Zeev Y, Bonevski B, Lim LL (2019). Improving health providers smoking cessation care in pregnancy: a systematic review and meta-analysis. Addict Behav.

[23] Fiore MC, Baker TB (2011). Treating smokers in the health care setting. N Engl J Med.

[24] Agency for Healthcare Research and Quality (2012). Five Major Steps to Intervention (The “5 A’s”).

[25] Fagerström KO (1978). Measuring degree of physical dependence to tobacco smoking with reference to individualization of treatment. Addict Behav.

[26] Skivington K, Matthews L, Simpson SA (2021). A new framework for developing and evaluating complex interventions: update of Medical Research Council guidance. BMJ.

[27] Cambon L, Terral P, Alla F (2019). From intervention to interventional system: towards greater theorization in population health intervention research. BMC Public Health.

[28] Hawe P, Shiell A, Riley T (2009). Theorising interventions as events in systems. Am J Community Psychol.

[29] Arksey H, O’Malley L (2005). Scoping studies: towards a methodological framework. Int J Soc Res Methodol.

[30] Page MJ, McKenzie JE, Bossuyt PM (2021). The PRISMA 2020 statement: an updated guideline for reporting systematic reviews. BMJ.

[31] Legido-Quigley H, McKee M, Nolte E, Glinos IA (2008). Assuring the quality of health care in the European Union: A case for action.

[32] Li M, Okamoto R, Tada A, Kiya M (2020). Factors associated with prenatal smoking cessation interventions among public health nurses in Japan. Int J Environ Res Public Health.

[33] Gould GS, Twyman L, Stevenson L (2019). What components of smoking cessation care during pregnancy are implemented by health providers? A systematic review and meta-analysis. BMJ Open.

[34] Baraona LK, Lovelace D, Daniels JL, McDaniel L (2017). Tobacco harms, nicotine pharmacology, and pharmacologic tobacco cessation interventions for women. J Midwifery Womens Health.

[35] Colomar M, Tong VT, Morello P (2015). Barriers and promoters of an evidenced-based smoking cessation counseling during prenatal care in Argentina and Uruguay. Matern Child Health J.

[36] Passey ME, Longman JM, Adams C, Johnston JJ, Simms J, Rolfe M (2020). Factors associated with provision of smoking cessation support to pregnant women - a cross-sectional survey of midwives in New South Wales, Australia. BMC Pregnancy Childbirth.

[37] Chertok IR, Archer SH (2015). Evaluation of a midwife- and nurse-delivered 5 A’s prenatal smoking cessation program. J Midwifery Womens Health.

[38] De Wilde K, Tency I, Steckel S, Temmerman M, Boudrez H, Maes L (2015). Which role do midwives and gynecologists have in smoking cessation in pregnant women? - A study in Flanders, Belgium. Sex Reprod Healthc.

[39] Longman JM, Adams CM, Johnston JJ, Passey ME (2018). Improving implementation of the smoking cessation guidelines with pregnant women: how to support clinicians?. Midwifery.

[40] Zeev YB, Bonevski B, Twyman L (2017). Opportunities missed: a cross-sectional survey of the provision of smoking cessation care to pregnant women by Australian general practitioners and obstetricians. Nicotine Tob Res.

[41] Agaku IT, Olaiya O, Quinn C (2015). A mixed-methods assessment of a brief smoking cessation intervention implemented in Ohio Public Health Clinics, 2013. Matern Child Health J.

[42] Bailey BA (2015). Effectiveness of a pregnancy smoking intervention: the Tennessee intervention for pregnant smokers program. Health Educ Behav.

[43] Murphy K, Steyn K, Mathews C (2016). The midwife’s role in providing smoking cessation interventions for pregnant women: the views of midwives working with high risk, disadvantaged women in public sector antenatal services in South Africa. Int J Nurs Stud.

[44] Reeks R, Padmakumar G, Andrew B, Huynh D, Longman J (2019). Barriers and enablers to implementation of antenatal smoking cessation guidelines in general practice. Aust J Prim Health.

[45] Skivington K, Matthews L, Simpson SA (2021). Framework for the development and evaluation of complex interventions: gap analysis, workshop and consultation-informed update. Health Technol Assess.

[46] Provost MH, Moreault L, Cardinal L (2016). Description, impact et conditions d’efficacité des stratégies visant l’intégration de la prévention dans les pratiques cliniques: Revue de la littérature, mise à jour 2007-2014.

[47] Moore G, Campbell M, Copeland L (2021). Adapting interventions to new contexts-the ADAPT guidance. BMJ.

[48] Senn N, Breton M, Ebert ST, Lamoureux-Lamarche C, Lévesque JF (2021). Assessing primary care organization and performance: literature synthesis and proposition of a consolidated framework. Health Policy.

[49] Tong EK, Strouse R, Hall J, Kovac M, Schroeder SA (2010). National survey of U.S. health professionals’ smoking prevalence, cessation practices, and beliefs. Nicotine Tob Res.

[50] Tremblay M, Cournoyer D, O’Loughlin J (2009). Do the correlates of smoking cessation counseling differ across health professional groups?. Nicotine Tob Res.

[51] Martínez C, Castellano Y, Andrés A (2017). Factors associated with implementation of the 5A’s smoking cessation model. Tob Induc Dis.

