# Supplementary material for: Factors associated with the implementation of the 5As model of smoking cessation support during pregnancy: A scoping review
Source: Tob Induc Dis. 2023 Aug 30;21:110. doi: 10.18332/tid/169623 (PMC10467347; doi:10.18332/tid/169623)
Supplement: Supplementary file 1 [file TID-21-110-s1.pdf]

**Supplementary Material 1 : Description of included articles**

| Ref. | Authors | Title | Date | Location | Aim related to our scoping review | Methods | Settings |
|---|---|---|---|---|---|---|---|
| 32 | Li M, Okamoto R, Tada A, Kiya M. | Factors Associated with Prenatal Smoking Cessation Interventions among Public Health Nurses in Japan. | 2020 | Japan | "To identify the factors associated with prenatal smoking cessation interventions based on the 5As model" | Quantitative study | Health centers of municipalities and special wards |
| 33 | Gould GS, Twyman L, Stevenson L, Gribbin GR, Bonevski B, Palazzi K, Bar Zeev Y. | What components of smoking cessation care during pregnancy are implemented by health providers? A systematic review and meta-analysis. | 2019 | Australia | "to examine which factors were associated with delivery of the 5As" | Systematic review and meta-analysis | Any setting |
| 34 | Baraona LK, Lovelace D, Daniels JL, McDaniel L. | Tobacco Harms, Nicotine Pharmacology, and Pharmacologic Tobacco Cessation Interventions for Women. | 2017 | United States of America | N/A | Review | N/A |
| 35 | Colomar M, Tong VT, Morello P, Farr SL, Lawsin C, Dietz PM, Aleman A, Berrueta M, Mazzoni A, Becu A, Buekens P, Belizán J, Althabe F. | Barriers and promoters of an evidenced-based smoking cessation counseling during prenatal care in Argentina and Uruguay. | 2015 | Argentina Uruguay | "To improve the understanding of systematic and individual factors influencing the implementation of the 5A's in prenatal care settings" | Qualitative study | Public prenatal clinics |
| 36 | Passey ME, Longman JM, Adams C, Johnston JJ, Simms J, Rolfe M. | Factors associated with provision of smoking cessation support to pregnant women - a cross-sectional survey of midwives in New South Wales, Australia. | 2020 | Australia | "To examine the association between midwives' self-reported | Quantitative study | Public health system |

| | | | | | | | |
|---|---|---|---|---|---|---|---|
| | | | | | implementation of the 5As and reported barriers and enablers to their implementation” | | |
| 37 | Chertok IR, Archer SH. | Evaluation of a midwife- and nurse-delivered 5 A's prenatal smoking cessation program. | 2015 | United States of America | “To evaluate the implementation of the ACOG 5 A’s program on prenatal care” | Quantitative study | Prenatal care clinics |
| 38 | De Wilde K, Tency I, Steckel S, Temmerman M, Boudrez H, Maes L. | Which role do midwives and gynecologists have in smoking cessation in pregnant women? - A study in Flanders, Belgium. | 2015 | Belgium | “To explore the implementation of a smoking cessation policy” | Qualitative study | Prenatal care |
| 39 | Longman JM, Adams CM, Johnston JJ, Passey ME | Improving implementation of the smoking cessation guidelines with pregnant women: How to support clinicians? | 2018 | Australia | “To explore the enablers and barriers to implementation of the antenatal smoking cessation guidelines among clinicians” | Qualitative study | Public health system |
| 40 | Zeev Y, Bonevski B, Twyman L, Watt K, Atkins L, Palazzi K, Oldmeadow C, Gould GS. | Opportunities Missed: A Cross-Sectional Survey of the Provision of Smoking Cessation Care to Pregnant Women by Australian General Practitioners and Obstetricians. | 2017 | Australia | “To examine (…) barriers and enablers to [smoking cessation care]” | Quantitative study | N/A |
| 41 | Agaku IT, Olaiya O, Quinn C, Tong VT, Kuiper NM, Conrey EJ, | A Mixed-Methods Assessment of a Brief Smoking Cessation Intervention Implemented in Ohio Public Health Clinics, 2013. | 2015 | United States of America | To assess “barriers and facilitators to implementing the 5As" | Mixed-methods approach | Public health clinics |

| | | | | | | | |
|---|---|---|---|---|---|---|---|
| | Sharma AJ, Mullen S, Dee D. | | | | | | |
| 42 | Bailey BA. | Effectiveness of a Pregnancy Smoking Intervention: The Tennessee Intervention for Pregnant Smokers Program. | 2015 | United States of America | "To describe the components of a smoking cessation intervention" | Quantitative study | Prenatal practices |
| 43 | Murphy K, Steyn K, Mathews C. | The midwife's role in providing smoking cessation interventions for pregnant women: The views of midwives working with high risk, disadvantaged women in public sector antenatal services in South Africa. | 2016 | South Africa | "To assess current attitudes and practices of midwives in relation to smoking education or counselling with pregnant women and the possible factors which would facilitate or hinder their participation in a potential smoking cessation intervention" | Mixed-methods approach | Maternal Obstetric Units |
| 44 | Reeks R, Padmakumar G, Andrew B, Huynh D, Longman JM. | Barriers and enablers to implementation of antenatal smoking cessation guidelines in general practices | 2019 | Australia | "To identify and understand the facilitators and barriers to providing smoking cessation support in antenatal care perceived by GPs" | Qualitative study | Antenatal care |

**Supplementary Material 2: Factors associated with the implementation of the 5As model in pregnancy**

| Theoretical framework | Categories | Sub-categories | Factors | Li et al.[32] | Gould et al.[33] | Baraona et al.[34] | Colomar et al.[35] | Passey et al.[36] | Chertok et al.[37] | De Wild et al.[38] | Longman et al.[39] | Zeev et al.[40] | Agaku et al.[41] | Bailey[42] | Murphy et al.[43] | Reeks et al.[44] |
|---|---|---|---|---|---|---|---|---|---|---|---|---|---|---|---|---|
| Clinical level | Clinical practices | Posture | Has a trusting relationship and positive attitude towards the pregnant woman | | | X | X | | X | | | | | | X | |
| | | | Takes into account the pregnant woman as a whole | | | X | | | X | | | | | | | X |
| | | | Takes a pro-active approach during every contact with the pregnant woman | | | X | | X | | | | | | | | |
| | | Interprofessional cooperation | Builds a interprofessional partnership | | | | | | X | | | | | | | |
| | | | Establishes communication between stakeholders (professionals, partners) | | | | X | | X | | | | | | | |
| | | | Knows the network of local actors | | | | | X | | | | | X | | | |
| | | clinical support tools | Uses motivational interviewing | | X | X | | | | X | | | | | X | |
| | | | Produces informatial materials for pregnant women | | X | | X | X | | | | | X | | X | |
| Clinical level | Perceptions of the 5As model | | Takes into account the doubts of professionals about the effectiveness of the 5As model | | | X | | | | | | X | X | | | |
| | | | Provides information on the effectiveness of referring | | | | | | | X | | | | | | |

| | | | | | | | | | | | | | | | | |
|---|---|---|---|---|---|---|---|---|---|---|---|---|---|---|---|---|
| **Clinical level** | **Professional role and identity** | Perceived role | Clarifies the importance of tobacco management during pregnancy follow-up | | | | | X | | | | X | | | X | |
| | | | Encourages professionals to embrace their role | | | | X | | | | | | | | X | X |
| | | Motivation | Builds on the interest and motivation of professionals for the 5As model and its implementation | | | | X | | | | X | | | X | | |
| | | | Builds on the willingness of professionals to change smoking behavior | | | | X | | | | | | | | | X |
| | | | Pays particular attention to the fact that gynecologists do not feel concerned by prevention | | | | | | | X | X | | | | | |
| | | | Involves midwives and nurses | | | | X | | | | | | | | | |
| | | Self-efficacy | Improves the self-efficacy of professionals | X | | X | X | | | | | X | X | | X | X |
| | | | Improves the legitimacy of professionals regardless of their personal smoking status | | | | X | | | | | | | | | |
| **Clinical level** | **Health professionals' perceptions of pregnant women** | | Takes into account the perception of smoking as a choice | | | | | | | | X | | X | | | |
| | | | Takes into account the negative perception of the pregnant woman | | | | | | | X | | | | | X | |
| | | | Takes into account the fear of losing the relationship with the pregnant woman | | | X | X | | | | | | | | | X |
| **Clinical level** | **Influence of beneficiaries** | | Takes into account the lack of knowledge about the health effects of tobacco on women and children | | | | X | | | | | | X | | X | |
| | | | Takes into account the lack of motivation of pregnant women | | | | | | | | | | | | X | |

| | | | | | | | | | | | | | | | | |
|---|---|---|---|---|---|---|---|---|---|---|---|---|---|---|---|---|
| | | | Takes into account the poor estimation of the difficulty of smoking cessation | | | | X | | | | | | | | | |
| | | | Takes into account the dissatisfaction of pregnant women with professionals | | | | X | | | | | | | | | |
| **Organization level** | **Organization of practices** | Healthcare pathway | Uses time in the waiting room for smoking prevention | | | | X | | | | | | | | | |
| | | | Uses time during the examination for smoking prevention | | | | | | | | X | | | | | |
| | | | Integrates tobacco cessation into pre-existing consultations | | | | X | | | | | | X | | | |
| | | | Offers consultations dedicated to tobacco management | | | X | | | | | | | | | | X |
| | | Deployment | Develops a 5As model deployment protocol | | | | X | X | | | | | | | | |
| | | | Involves line management in communication in relation to the 5As model | | | | | | | | X | | X | | | |
| | | | Involves opinion leaders in the communication of the 5As model | | | | X | X | | | X | | | | | |
| | | | Sets up a monitoring of the implementation of the 5As model | | | | | X | | | | | | | | |
| **Organization level** | **Resources** | Financial resources | Takes into account the cost of implementation | | | X | X | | X | | | X | | X | | |
| | | Time resources | Takes into account the time available | | | X | X | X | X | X | | X | X | X | X | X |

| | | | | | | | | | | | | | | | | |
|---|---|---|---|---|---|---|---|---|---|---|---|---|---|---|---|---|
| | | | Prioritizes the issues to be addressed during perinatal consultations | | | | | | | | X | | | | | |
| | | | Takes into account the patient's comorbidities | | | X | | | | | | | | | | |
| **Organization level** | **Support for professionals** | Training | Follows a training on the 5As method | X | X | X | X | X | X | X | | X | X | X | X | |
| | | | Acquires skills (e.g. having a positive posture towards the woman's smoking history, communicating on the risks for the fetus, implementing each step of the 5As model) | | | | X | | | | X | | X | | | X |
| | | | Acquires knowledge (e.g. on NRTs, on addictions and on the specific risks of tobacco on the fetus) | | | | X | | | X | X | | X | | X | X |
| | | Resources for professionals | Develops a clinical guidelines | | X | | X | | | | | | | | | |
| | | | Develops and disseminates expert recommendations | | | | X | | | | | | X | | | |
| | | | Sets up automatic computer reminders | | X | | X | X | | | X | | | | | |
| | | | Trains professionals in the 5As model to support perinatal professionals | | X | | | | X | | | | | | | |
| | | | Provides feedback on the practices of professionals | | | | X | | | | | | | | | |
| **Health system level** | **Political environment** | | Involves patient organizations (advocacy) | | | | | X | | | | | | | | |
| | | | Has commitment from management | | | X | X | | | | | | | | | |

| | | | | | | | | | | | | | |
|---|---|---|---|---|---|---|---|---|---|---|---|---|---|
| Makes smoking cessation a priority (support from management teams, authorities, learned societies, etc.) | | | X | | | | | | | | | | |

## Supplementary Material 3: PRISMA 2020 checklist

| Section and Topic | Item # | Checklist item | Location where item is reported |
|---|---|---|---|
| **TITLE** | | | |
| Title | 1 | Identify the report as a systematic review. | P.1 |
| **ABSTRACT** | | | |
| Abstract | 2 | See the PRISMA 2020 for Abstracts checklist. | |
| **INTRODUCTION** | | | |
| Rationale | 3 | Describe the rationale for the review in the context of existing knowledge. | P. 2-3 |
| Objectives | 4 | Provide an explicit statement of the objective(s) or question(s) the review addresses. | p.3 |
| **METHODS** | | | |
| Eligibility criteria | 5 | Specify the inclusion and exclusion criteria for the review and how studies were grouped for the syntheses. | p.3 |
| Information sources | 6 | Specify all databases, registers, websites, organisations, reference lists and other sources searched or consulted to identify studies. Specify the date when each source was last searched or consulted. | p.3 |
| Search strategy | 7 | Present the full search strategies for all databases, registers and websites, including any filters and limits used. | p.3-4 |
| Selection process | 8 | Specify the methods used to decide whether a study met the inclusion criteria of the review, including how many reviewers screened each record and each report retrieved, whether they worked independently, and if applicable, details of automation tools used in the process. | p.3-4 |
| Data collection process | 9 | Specify the methods used to collect data from reports, including how many reviewers collected data from each report, whether they worked independently, any processes for obtaining or confirming data from study investigators, and if applicable, details of automation tools used in the process. | P.3-4 |
| Data items | 10a | List and define all outcomes for which data were sought. Specify whether all results that were compatible with each outcome domain in each study were sought (e.g. for all measures, time points, analyses), and if not, the methods used to decide which results to collect. | Not concerned |
| | 10b | List and define all other variables for which data were sought (e.g. participant and intervention characteristics, funding sources). Describe any assumptions made about any missing or unclear information. | p. 3-4 |

| Section and Topic | Item # | Checklist item | Location where item is reported |
|---|---|---|---|
| Study risk of bias assessment | 11 | Specify the methods used to assess risk of bias in the included studies, including details of the tool(s) used, how many reviewers assessed each study and whether they worked independently, and if applicable, details of automation tools used in the process. | Not concerned |
| Effect measures | 12 | Specify for each outcome the effect measure(s) (e.g. risk ratio, mean difference) used in the synthesis or presentation of results. | Not concerned |
| Synthesis methods | 13a | Describe the processes used to decide which studies were eligible for each synthesis (e.g. tabulating the study intervention characteristics and comparing against the planned groups for each synthesis (item #5)). | p.3 |
| | 13b | Describe any methods required to prepare the data for presentation or synthesis, such as handling of missing summary statistics, or data conversions. | Not concerned |
| | 13c | Describe any methods used to tabulate or visually display results of individual studies and syntheses. | Not concerned |
| | 13d | Describe any methods used to synthesize results and provide a rationale for the choice(s). If meta-analysis was performed, describe the model(s), method(s) to identify the presence and extent of statistical heterogeneity, and software package(s) used. | p.3-4 |
| | 13e | Describe any methods used to explore possible causes of heterogeneity among study results (e.g. subgroup analysis, meta-regression). | Not concerned |
| | 13f | Describe any sensitivity analyses conducted to assess robustness of the synthesized results. | Not concerned |
| Reporting bias assessment | 14 | Describe any methods used to assess risk of bias due to missing results in a synthesis (arising from reporting biases). | p.4 |
| Certainty assessment | 15 | Describe any methods used to assess certainty (or confidence) in the body of evidence for an outcome. | Not concerned |
| **RESULTS** | | | |
| Study selection | 16a | Describe the results of the search and selection process, from the number of records identified in the search to the number of studies included in the review, ideally using a flow diagram. | |
| | 16b | Cite studies that might appear to meet the inclusion criteria, but which were excluded, and explain why they were excluded. | p.4 fig.1 |
| Study characteristics | 17 | Cite each included study and present its characteristics. | p.4 Supp. mat. 1 |
| Risk of bias in studies | 18 | Present assessments of risk of bias for each included study. | Not concerned |
| Results of | 19 | For all outcomes, present, for each study: (a) summary statistics for each group (where appropriate) and (b) an effect estimate and | p. 4-7 |

| Section and Topic | Item # | Checklist item | Location where item is reported |
|---|---|---|---|
| individual studies | | its precision (e.g. confidence/credible interval), ideally using structured tables or plots. | Supp. mat 2 |
| Results of syntheses | 20a | For each synthesis, briefly summarise the characteristics and risk of bias among contributing studies. | Supp. mat. 1 |
| | 20b | Present results of all statistical syntheses conducted. If meta-analysis was done, present for each the summary estimate and its precision (e.g. confidence/credible interval) and measures of statistical heterogeneity. If comparing groups, describe the direction of the effect. | Not concerned |
| | 20c | Present results of all investigations of possible causes of heterogeneity among study results. | Not concerned |
| | 20d | Present results of all sensitivity analyses conducted to assess the robustness of the synthesized results. | Not concerned |
| Reporting biases | 21 | Present assessments of risk of bias due to missing results (arising from reporting biases) for each synthesis assessed. | Not concerned |
| Certainty of evidence | 22 | Present assessments of certainty (or confidence) in the body of evidence for each outcome assessed. | Not concerned |
| **DISCUSSION** | | | |
| Discussion | 23a | Provide a general interpretation of the results in the context of other evidence. | p. 8-10 |
| | 23b | Discuss any limitations of the evidence included in the review. | p. 9-10 |
| | 23c | Discuss any limitations of the review processes used. | p. 9-10 |
| | 23d | Discuss implications of the results for practice, policy, and future research. | p. 8-10 |
| **OTHER INFORMATION** | | | |
| Registration and protocol | 24a | Provide registration information for the review, including register name and registration number, or state that the review was not registered. | Not concerned |
| | 24b | Indicate where the review protocol can be accessed, or state that a protocol was not prepared. | Not concerned |
| | 24c | Describe and explain any amendments to information provided at registration or in the protocol. | Not concerned |
| Support | 25 | Describe sources of financial or non-financial support for the review, and the role of the funders or sponsors in the review. | p. 10 |
| Competing interests | 26 | Declare any competing interests of review authors. | p. 11 |
| Availability of | 27 | Report which of the following are publicly available and where they can be found: template data collection forms; data extracted from | Not |

| Section and Topic | Item # | Checklist item | Location where item is reported |
|---|---|---|---|
| data, code and other materials | | included studies; data used for all analyses; analytic code; any other materials used in the review. | concerned |

© 2023 Burtin A. et al.
